# Ear malformation in a child with Goldenhar syndrome and its appropriate audiological management

**DOI:** 10.23938/ASSN.1102

**Published:** 2025-02-13

**Authors:** Andrés González Fernández, Manuela del Carmen Zapata, José Zubicaray Ugarteche

**Affiliations:** University Hospital of Navarra Otolaryngology and Head and Nek Surgery Department Pamplona Spain

**Keywords:** Goldenhar Disease, Audiology, Middle ear, External ear, Child, Síndrome de Goldenhar, Audiología, Oído Medio, Oído externo, Niño

## Abstract

Goldenhar syndrome is a rare congenital disorder characterized by defects in the development of structures derived from the first and the second branchial arches. This condition encompasses a range of symptoms, including craniofacial, ocular, vertebral, and auricular abnormalities.

We present the case of a 6-year-old girl with right temporal bone hypoplasia and preauricular tag from birth, leading to a diagnosis of Goldenhar syndrome. She exhibited various middle and external ear defects, and her audiological treatment was crucial in ensuring optimal neurological and speech development. In adolescence, if the Eustachian tube remains stable, surgical repair of the ossicular chain may be considered.

## RESUMEN

El síndrome de Goldenhar es una enfermedad congénita poco frecuente, caracterizada por el desarrollo defectuoso de estructuras derivadas del primer y segundo arco branquial. Por tanto, presenta una amplia gama de síntomas que incluyen anomalías craneofaciales, oculares, vertebrales y auriculares.

Se presenta el caso de una niña de 6 años con hipoplasia del hueso temporal derecho y un apéndice preauricular desde el nacimiento, por lo que se le diagnosticó síndrome de Goldenhar. Presentaba algunos defectos del oído medio y externo, y su tratamiento audiológico permitió garantizar su óptimo desarrollo neurológico y del habla. A partir de la adolescencia, y si la trompa de Eustaquio es estable, se reconsiderará la opción de cirugía reparadora de la cadena de huesecillos.

## INTRODUCTION

Goldenhar syndrome is a rare multifactorial condition characterized by defects in the development of structures derived from the first and the second branchial arches^1^^-^^5^. This syndrome encompasses a variety of symptoms, including craniofacial, ocular, vertebral, and auricular abnormalities^1^^,^^2^^,^^4^^-^^6^. In some cases, it may also affect visceral organs, including the cardiac, renal, and nervous systems^1^^,^^6^^,^^7^. The auricular abnormalities primarily involve external and middle ear defects, although inner ear malformations have also been reported^2^^-^^4^^,^^6^^,^^7^. As a result, individuals with Goldenhar syndrome often experience varying degrees of hearing loss, predominantly conductive in nature^2^.

We present the case of a 6-year-old girl with right temporal bone hypoplasia and preauricular tag from birth, diagnosed with the rare Goldenhar syndrome. Early diagnosis and appropriate management of audiological defects are crucial to ensure optimal neurological and speech development.

## CASE REPORT

We present the case of a 6-year-old girl with right temporal bone hypoplasia and preauricular tag from birth.

During neonatal examination, a systolic murmur was detected. As a result, a cardiologist diagnosed an abnormal interventricular communication without hemodynamic repercussions. The neonatal hearing test, using otoacoustic emissions, revealed a defect in the right ear, and moderate hearing loss was confirmed with the auditory steady-state response. Due to the hearing loss, a magnetic resonance imaging study was requested, which showed no abnormalities along the auditory pathways.

A few months after, the preauricular tag was surgically removed. During the same act, an additional auditory steady-state response study was performed, revealing no changes compared to the neonatal assessment. Simultaneously, ophthalmologists investigated the possibility of an epibulbar dermoid and visual defects, in accordance with the diagnostic criteria for Goldenhar syndrome. However, the ophthalmological examination was normal. Additionally, both the kidneys and nervous system were evaluated to identify any other potential abnormalities, but no defects were found.

**Figure 1:**
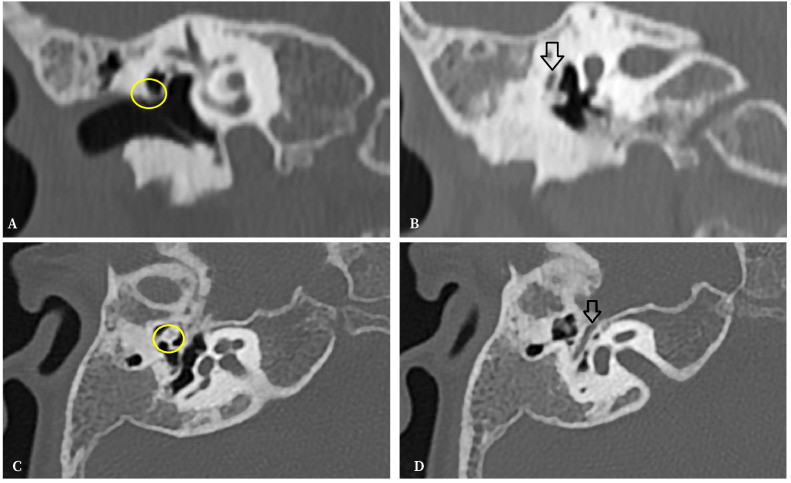
Computed tomography. **A**. Coronal slice. Hypoplasia of the tympanic cavity and the defect in ossicular chain with the incus attached to the tympanic wall (circle). **B**. Coronal slice. Abnormal course of the facial nerve (arrow). **C**. Axial slice. Hypoplasia of the tympanic cavity and the ossicular chain with fixation of incus to the tympanic wall (circle), and lack of mastoid pneumatisation. **D**. Axial slice. Abnormal course of facial nerve (arrow).

With the diagnosis of Goldenhar syndrome, suspecting a conductive hearing loss and with no effusion inside the middle ear, a computed tomography study was requested. The last showed hypoplasia of the tympanic cavity and the ossicular chain with the incus attached to the tympanic wall and an abnormal course of the facial nerve (Figura 1).

Due to the instability of the Eustachian tube in children and the potential for an unsuccessful ossiculoplasty, surgical treatment was deemed unfeasible. Instead, a right ear hearing aid was recommended.

**Figure 2:**
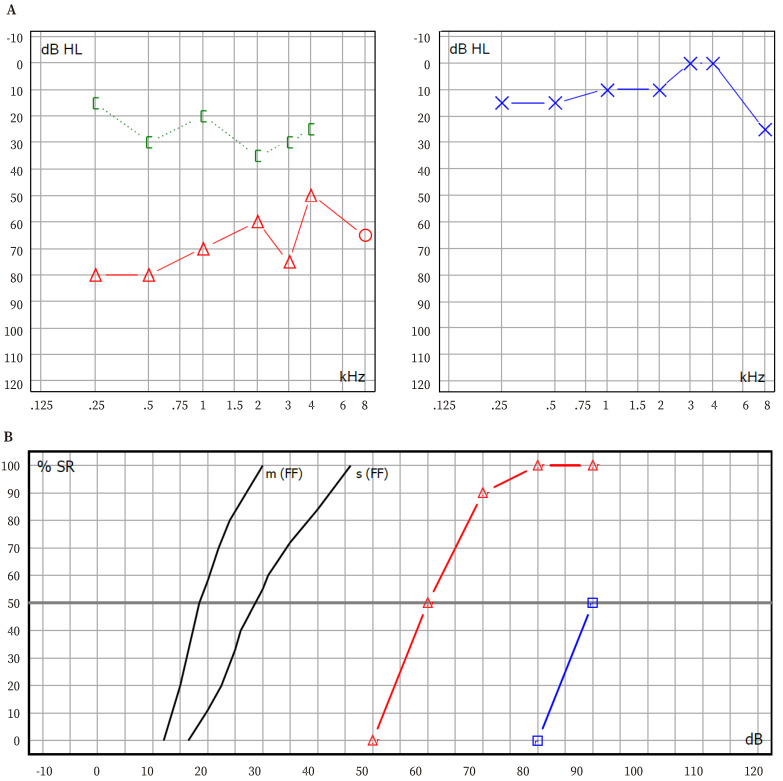
Audiometry. **A.** Mixed and moderate right hearing loss. Blue line represents the air way in the left ear. Red line and green line represent air way and bone way in the right ear. **B.** Benefit of hearing aid. The verbal threshold (intensity required for listening half of the words of the proof) improves from 90 dB without hearing aid (blue line) to 60 dB with the hearing aid (red line), and also, the patient achieves 100% of total discrimination.

At the age of five, conventional audiometry was performed (Figura 2A), revealing mixed, moderate hearing loss. With the hearing aid, the benefit was significant, with an improvement in the verbal perception threshold from 90 dB to 60 dB (this threshold represents the intensity required to correctly hear 50% of the words in the test), achieving 100% word discrimination (Figura 2B).

Currently, biannual follow-up visits are scheduled. In adolescence, if the Eustachian tube remains stable, we will reassess the possibility of surgical repair of the ossicular chain.

## DISCUSSION

Goldenhar syndrome, also known as oculo- auriculo-vertebral dysplasia, is characterized by defects in the development of structures derived from the first and the second branchial arches^1^^-^^3^^,^^5^. Its incidence ranges from 1 in 5,300 to 1 in 260,500 live births^4^^,^^5^^,^^7^, with a higher prevalence in boys than in girls^4^. Additionally, the right side of the face is more frequently affected^(8)^, as observed in the patient described here.

Currently, the diagnostic criteria proposed by the International Consortium for Health Outcomes Measurements are recommended for evaluating the abnormalities associated with this syndrome. In essence, diagnosis requires the presence of at least two major criteria (such as mandibular hypoplasia, microtia, orbital/facial bone hypoplasia, or asymmetric facial movement), at least one major and one minor criteria (including facial soft tissue deficiency, preauricular tags, macrostomia, clefting, epibulbar dermoids, hemivertebrae), or at least three minor criteria^5^. The patient described in this report exhibits temporal bone hypoplasia (a major criterion) and a preauricular tag (a minor criterion), thus confirming the diagnosis.

Abnormal development in Goldenhar syndrome may result from disruption in the migration of mesodermal o neural crest cells, or from abnormal embryonic vascular supply to the affected branchial arches^1^^,^^2^^,^^6^^,^^7^. The exact aetiology remains unknown; however, it is suggested that genetic abnormalities in combination with certain environmental factors, may contribute to the condition. These factors include the administration of retinoic acid, thalidomide, oral anticoagulants, vitamin A derivatives, tamoxifen, cocaine, and/or alcohol during pregnancy^1^^,^^2^^,^^6^. Gestational diabetes mellitus has also been investigated as a potential risk factor^2^, as has vaginal bleeding during the second trimester^7^. To the best of our knowledge, none of these known risk factors was present in the case reported here.

The structural abnormalities associated with hearing loss in Goldenhar syndrome involve defects in various structures of the external, middle, and inner ear^2^^,^^3^^,^^4^ (Table 1). As a result, hearing loss is common in individuals with this syndrome^2^^,^^4^.

Defects in the external and middle ear are more common than abnormalities in the inner ear in Goldenhar syndrome. As a result, hearing loss is typically conductive^2^^,^^4^. However, some reported cases also include defects in the inner ear, leading to sensorineural or mixed hearing loss^2^^-^^4^. The current explanation for inner ear involvement is related to a failure in the migration of neural crest cells^2^^,^^4^.

**Table 1:** Frequent ear defects in Goldenhar syndrome

Ear’s area	Defects
External	- Hypoplasia of the pinna (some degrees)
	- Stenosis or absence of the external auditory canal
	- Preauricular outgrowths
Middle	- Hypoplasia of the tympanic membrane
	- Hypoplasia of tympanic cavity
	- Hypoplasia and lack of auditory ossicles
	- Improper course of the facial nerve
	- Lack of tensor tympani muscle
	- Lack of chorda tympani
	- Lack of oval and round windows
Inner	- Cochlear hypoplasia
	- Absence of cochlear aqueduct
	- Immature vestibular system
	- Absence of the semicircular canals
	- Enlargement of the vestibular aqueduct
	- Absence of the facial nerve
	- Internal auditory canal defects

Audiological management varies depending of the specific ear defect. For atresia of external auditory canal, the treatment of choice is a bone-anchored implant. If the ossicular chain is malformed, ossiculoplasty may be considered. In cases of sensorineural hearing loss, conventional hearing aid can be an effective solution. However, for severe or profound deafness, cochlear implants are often the best option^2^. In the case of our patient, a conventional hearing aid is currently used due to Eustachian tube instability during childhood and the potential risk of ossiculoplasty failure. However, we will reassess the possibility of surgical repair of the ossicular chain during adolescence if the Eustachian tube stabilizes.

In conclusion, given the variety of otologic abnormalities that may be present in Goldenhar syndrome, it is crucial to identify them early and assess hearing function. Tailored strategies should be employed based on the type and severity of the hearing loss and the underlying anatomical defects. Selecting the appropriate treatment for audiological defects will help the child achieve optimal auditory function, supporting speech and neurological development.

## Data Availability

They are available upon request to the corresponding author.
